# Environmental persistence and transmission dynamics of *Serratia* AS1 in mosquito habitats: advancing paratransgenesis for malaria control

**DOI:** 10.1128/aem.01840-25

**Published:** 2025-11-25

**Authors:** Hossein Kamel Urmia, Mona Koosha, Bagher Yakhchali, Seyed Hassan Moosa-Kazemi, Mohammad Ali Oshaghi

**Affiliations:** 1 Department of Vector Biology and Control of Diseases, School of Public Health, Tehran University of Medical Sciences 48439, Tehran, Iran; 2 Department of Medical Parasitology and Mycology, School of Medicine, Shahid Beheshti University of Medical Sciences 556492, Tehran, Iran; 3 Institute of Industrial and Environmental Biotechnology, National Institute of Genetic Engineering and Biotechnology, Tehran, Iran; Universite de la Reunion, Ste Clotilde, France

**Keywords:** malaria control, paratransgenesis, *Serratia *AS1, *Anopheles stephensi*, semi-field study

## Abstract

Malaria remains a major global health challenge, particularly in developing countries, necessitating innovative control strategies. With rising resistance of *Plasmodium* to drugs and *Anopheles* mosquitoes to insecticides, paratransgenesis—using engineered symbiotic bacteria to deliver anti-pathogen molecules—offers a promising alternative. Translating this approach to field applications requires rigorous evaluation under semi-field conditions. We evaluated the environmental stability and transmission dynamics of *Serratia* AS1-mCherry, a paratransgenesis candidate, in *Anopheles stephensi* habitats under semi-field conditions in Bandar Abbas, Iran. *Serratia* AS1 successfully colonized mosquito midguts and ovaries, persisted in larval breeding water for 14 days, and remained stable on sugar-soaked cotton pads for 4–6 days. Transmission routes include transstadial, venereal, and vertical transmission, in addition to adult acquisition from larval habitats (sipping), demonstrating robust colonization and dissemination. Water-based delivery effectively disseminates *Serratia* AS1 among mosquito populations, highlighting its potential for paratransgenesis-based malaria control. This study establishes the feasibility of using *Serratia* AS1 with effector molecules in field settings, offering a sustainable strategy for managing vector-borne diseases.

**IMPORTANCE:**

Malaria remains a major health challenge, especially in developing countries where traditional control methods like insecticides and drugs are becoming less effective due to resistance. This study explores a promising new approach called paratransgenesis, which uses genetically modified bacteria to fight malaria. We tested a bacterium called *Serratia* AS1, which can live inside mosquitoes and spread through their populations. Our experiments showed that *Serratia* AS1 can survive in mosquito breeding sites and spread effectively among mosquitoes through multiple routes, such as larval water, sugar sources, and even from parent mosquitoes to their offspring. These findings suggest that *Serratia* AS1 could be used to deliver anti-malaria molecules to mosquitoes in the wild, offering a sustainable and innovative way to control the disease. This work brings us one step closer to using paratransgenesis as a practical tool to reduce malaria transmission and save lives.

## INTRODUCTION

Malaria remains a pervasive parasitic disease, affecting approximately 140 countries across tropical and subtropical regions. It is caused by protozoan parasites of the genus *Plasmodium*, transmitted through the bites of female *Anopheles* mosquitoes. According to the World Health Organization (WHO), an estimated 263 million malaria cases were reported globally in 2023, leading to over 597,000 deaths, with children being disproportionately affected (1). Current control strategies focus on timely diagnosis, effective treatment, and vector management through insecticide-treated bed nets (ITNs) and indoor residual spraying (IRS). However, the growing resistance of both *Plasmodium* to antimalarial drugs (2) and *Anopheles* mosquitoes to insecticides (3) has necessitated the development of alternative approaches.

Paratransgenesis represents an innovative strategy for managing vector-borne diseases by leveraging genetically modified symbiotic or commensal microorganisms to disrupt pathogen transmission. Unlike traditional vector control methods, this approach focuses on exploiting the mosquito microbiota to inhibit parasite development within the vector (4). This is particularly advantageous since midgut bacteria proliferate significantly after a blood meal, placing them in close proximity to vulnerable parasite stages (5, 6). Following its initial demonstration in Chagas disease vectors using *Rhodococcus rhodnii* (7), paratransgenesis has been explored for malaria control, targeting the microbiota of key malaria vectors (5, 8–15).

*Serratia* sp., a facultative, aerobic, Gram-negative bacterium of the Yersiniaceae family, is a notable candidate for paratransgenesis. Naturally associated with various arthropods, including *Anopheles* mosquitoes (16–27), the *Serratia* AS1 strain exhibits an exceptional ability to colonize mosquito organs and proliferate within *Anopheles stephensi* and *Anopheles gambiae* populations (5). The *Serratia* AS1 strain was initially developed and characterized by the Jacobs-Lorena group, and its potential for paratransgenesis has been demonstrated in previous studies (5, 25, 28). Importantly, it minimally impacts mosquito fitness, with no significant adverse effects on lifespan, fecundity, or fertility (5, 25, 28). Genetically modified *Serratia* AS1 strains expressing anti-*Plasmodium* effector proteins have shown potential for reducing vectorial capacity (5).

The invasive malaria vector *Anopheles stephensi*, originally confined to South Asia, has expanded its range to multiple African countries, including Djibouti, Ethiopia, Sudan, Somalia, Nigeria, and Kenya, among others (1, 29–32). Its remarkable adaptation to urban settings and resistance to multiple insecticides, including those used in ITNs and IRS (33–42), present significant challenges to vector control. This highlights the urgent need for innovative and sustainable strategies to manage malaria transmission.

While paratransgenesis has shown promise in laboratory settings, the transition to field applications is hindered by regulatory and logistical challenges, including the effective delivery of modified bacteria to wild mosquito populations. This study addresses these challenges by evaluating the stability, transmission, and propagation of *Serratia* AS1-mCherry in *An. stephensi* under semi-field conditions in Bandar Abbas, Iran. We assessed the bacterium’s dynamics across mosquito developmental stages, including transstadial, venereal, and vertical transmission, as well as its persistence in larval breeding habitats and sugar solutions. This research provides critical insights into the ecological interactions of *Serratia* AS1 with mosquito hosts and its potential as a paratransgenesis candidate for malaria control.

## MATERIALS AND METHODS

### Study area

The study was conducted in Bandar Abbas, Hormozgan Province, southern Iran (27°11′46″N 56°17′16″E), a malaria-endemic area with a hot desert climate (BWh). Summer temperatures reach up to 49°C (120°F), while winter temperatures drop to 5°C (41°F). Annual rainfall is about 170 mm (6.7 in), with an average relative humidity of 65%. Bandar Abbas is Iran’s main seaport and a significant malaria-transmission focal point, with an urban population engaged primarily in trade, fishing, and port-related activities.

### Mosquito rearing conditions

The study utilized a hybrid population of *An. stephensi* mosquitoes to balance laboratory feasibility with field relevance. A laboratory colony of the Bandar Abbas strain—maintained for fewer than five generations to minimize adaptation to artificial conditions—was supplemented with wild mosquitoes collected from larval breeding sites in Bandar Abbas. The hybrid population was reared under standardized insectary conditions at 27 ± 1°C, 75 ± 5% relative humidity (RH), and a 12:12 h light-dark cycle. Larvae were reared in deionized water and fed daily with nutrient-rich pellet fish food. Rearing was conducted at the Anopheline insectarium, WHO Regional Malaria Training Center, Bandar Abbas Health Education and Research Station, Tehran University of Medical Sciences (43).

### Generation of *Serratia* AS1 expressing mCherry-fluorescent protein

The plasmid pBAM2-mCherry, carrying the mCherry-fluorescent protein gene, was electroporated into *Serratia* AS1 (courtesy of Dr. Marcelo Jacobs-Lorena, Department of Molecular Microbiology and Immunology, Johns Hopkins Bloomberg School of Public Health, USA) (5). This plasmid is maintained episomally and was not integrated into the bacterial genome. The modified strain, *Serratia* AS1-mCherry, was cultured and maintained at 28°C for all experiments.

### Preparing for bacterial suspension

*Serratia* AS1-mCherry was cultured in BHI broth at 28°C for 24–48 h until reaching OD_600_ = 1 (~10^9^ cells/mL). Bacterial cells were harvested by centrifugation at 3,000 × *g* for 10 min at 4°C, washed twice, and resuspended in sterile phosphate-buffered saline (PBS). For sugar bait preparation, the bacterial suspension was mixed with 5% sucrose solution containing 2.5% red food dye to achieve a final concentration of 10^9^ cells/mL.

### Semi-field set-up

**Fig 1:**
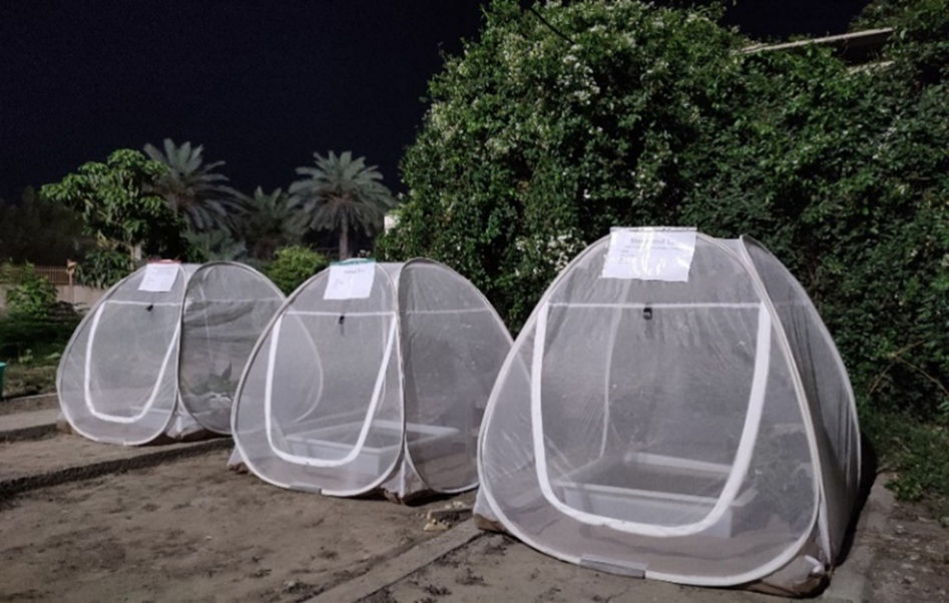
Semi-field set-up. Three pyramid-shaped cages (140 × 140 × 140 cm) were installed under field conditions. Inside each cage, PE trays (70 × 70 × 17 cm) filled with 2 cm–3 cm of garden soil and 40 L of deionized water were placed. Damp black pots and concrete blocks were provided for mosquito shelter and resting.

Three pyramid-shaped cages (140 × 140 × 140 cm) were installed in the research center garden. Inside each cage, polyethylene (PE) trays (70 × 70 × 17 cm) filled with 2 cm–3 cm of garden soil and 40 L of deionized water were placed (Fig. 1). The trays were elevated on sealed platforms and lined with waterproof barriers to prevent leakage. This design ensured physical containment of transgenic bacteria within the trays. To further minimize environmental dispersal, all experiments were conducted under biosafety level 2 protocols, including the use of dedicated equipment and restricted access to the cages.

After each experiment, the trays and the water and soil in the trays were treated with 10% sodium hypochlorite (bleach) for 24 h to sterilize transgenic bacteria, a method validated for eradicating bacteria. To verify containment, environmental swabs (*n* = 5 per cage) were collected weekly from the soil, water, and surfaces outside the trays and tested via culturing on brain-heart infusion (BHI) agar supplemented with kanamycin (100 µg/mL) (selection for the mCherry plasmid). Cultures were incubated at 28°C for 48–72 h, and no transgenic bacteria (mCherry-expressing colonies) were detected in any external samples.

### Surface sterilization and bacterial quantification

Surface sterilization and bacterial quantification were performed, respectively, on larvae/adult and water samples. Briefly, larvae/adults were transferred to microtubes containing 500 µL sterile water and kept on ice for 5 to 10 min. They were then transferred to tubes containing 500 µL of 10% sodium hypochlorite (NaClO) at 4°C for 10 min. After removing the NaClO, the samples were kept on ice for 5 min in 70% cold ethanol at −5°C to ensure surface sterilization. The specimens were then washed twice with PBS at 4°C. Individual specimens or pools of two to three sterilized specimens were homogenized in 300 µL PBS, and 100 µL of the homogenized solution was cultured on BHI agar supplemented with kanamycin (100 µg/mL) (selection for the mCherry plasmid). Fluorescent colonies were counted using either fluorescent microscopy or a normal stereomicroscope. For CFU analysis, each mosquito pool was considered a single experimental unit. The CFU count per mosquito was derived by dividing the total pool CFU by the number of mosquitoes in the pool. Statistical analysis was then performed on the means of these pools. To quantify the CFU per milliliter of *Serratia* AS1-mCherry in the larval habitat water, 10 mL of water was sampled. Samples were centrifuged at 3,000 × *g* for 10 min using an Eppendorf microcentrifuge. The pellet was resuspended in 1 mL of sterile PBS, and serial dilutions were prepared. A 100 µL aliquot of each dilution was plated onto BHI agar supplemented with kanamycin (100 µg/mL) and incubated at 28°C for 24–48 h. Colonies expressing mCherry fluorescence were enumerated under a stereomicroscope.

To verify surface sterilization, the washing PBS was examined for fluorescent bacteria. Experiments exhibiting contamination were excluded from the analysis. The experiments were repeated three times with similar results.

### Vertical transmission of *Serratia* AS1-mCherry

Female and male mosquitoes (2–5 days old) were maintained in 30 × 30 × 30 cm insectarium cages with *ad libitum* access to sterile 5% sucrose pads for 24 h prior to bacterial exposure. For bacterial acquisition, mosquitoes were provided with fresh sugar pads containing 10⁹ CFU/mL *Serratia* AS1-mCherry in 5% sucrose with 2.5% red food dye for 48 h. Bacterial ingestion was confirmed by visualizing red abdominal coloration using a stereomicroscope (40× magnification).

Following bacterial uptake, mosquitoes were transferred to 1.8 × 1.8 × 1.8 m semi-field cages containing white plastic oviposition trays (40 L dechlorinated sterile water). Mosquitoes received a single 30 min blood meal from an anesthetized rabbit (ketamine 30 mg/kg + xylazine 3 mg/kg–5 mg/kg, intramuscular). We monitored trays daily for egg deposition, subsequently collecting larval rearing and water sampling. Environmental conditions were maintained at 21°C–37°C and 15%–95% RH throughout the study period.

A total of 32 fourth-instar larvae, three habitat water samples, and 40 sugar-fed emerged females were processed across three independent replicates; the specific sample sizes and pooling strategy for each group are provided in Table 1.

**TABLE 1:** Sample size and pooling strategy for assessing *Serratia* AS1 transmission to *Anopheles stephensi* via different exposure routes^*a*^

Assay	Status	R	2nd-instar larva Pools	4th-instar larva Pools	P	4th-instar habitat water	Em. SF female Pools	Em. SF male pools	Spermatheca	Ovary	Midgut
Vertical	NA	1 2 3	NA	10 (2 × 3, 2 × 2) 11 (3 × 3, 1 × 2) 11 (3 × 3, 1 × 2)	NA	1 1 1	13 (3 × 3, 2 × 2) 13 (3 × 3, 2 × 2) 14 (4 × 3, 1 × 2)	NA	NA	NA	NA
Transstadial	NA	1 2 3	8 (2 × 3, 1 × 2) 11 (3 × 3, 1 × 2) 11 (3 × 3, 1 × 2)	8 (2 × 3, 1 × 2) 11 (3 × 3, 1 × 2) 11 (3 × 3, 1 × 2)	4 4 4	1 1 1	15 (3 × 3, 3 × 2) 17 (5 × 3, 1 × 2) 20 (6 × 3, 1 × 2)	3 (1 × 3) 3 (1 × 3) 6 (2 × 3)	NA	NA	NA
Venereal	SF female	1 2 3	NA	NA	NA	NA	NA	NA	10 9 8	6 8 7	5 8 7
	BF female	1 2 3	NA	NA	NA	NA	NA	NA	9 14 13	4 7 5	3 2 2
Sipping	Em. SF female	1 2 3	NA	NA	NA	NA	NA	NA	NA	NA	5 5 5
	Em. BF female	1 2 3	NA	NA	NA	NA	NA	NA	NA	NA	4 4 4

^*a*^ R, replicate; SF, sugar-fed; BF, blood-fed; P, pupa; Em, emerged adult**. **Values in the “pool” column represent the number of pools × the number of individuals per pool (e.g., 3 × 3 indicates three pools of three individuals each). NA, not applicable.

### Transstadial transmission of *Serratia* AS1-mCherry

To investigate potential transstadial transmission of *Serratia* AS1-mCherry, 300 second-instar *An. stephensi* larvae (100 larvae per replicate) were introduced into semi-field trays containing 40 L of larval breeding water inoculated with a bacterial suspension. The suspension was prepared by adding 5 mL of *Serratia* AS1-mCherry (1 × 10⁹ cells/mL) to the water, yielding a final concentration of 1.25 × 10⁵ cells/mL. Trays were maintained under semi-field conditions (22°C–37°C, 15%–95% RH).

To evaluate transstadial transmission of *Serratia* AS1-mCherry between larval stages, a subset of third-instar larvae (*n* = 30) underwent surface sterilization prior to molting to the fourth instar. Larvae were subjected to sequential washes in sterile deionized water (9 × 10 min immersions) to remove external bacterial contamination. After the final wash, larvae were transferred to 40 L of *Serratia-*free water and monitored until molting. To confirm sterility, post-wash water samples were plated on BHI agar supplemented with kanamycin (100 µg/mL). Despite sterilization efforts, *Serratia* AS1-mCherry colonies were detected, indicating persistent environmental contamination before and during molting.

At pupation, individuals were surface-sterilized to exclude external bacterial contamination. Pupae were washed sequentially in sterile deionized water (six to nine cycles of 10 min immersion) until culturing of the final wash water on BHI agar supplemented with kanamycin (100 µg/mL) confirmed no detectable *Serratia* AS1-mCherry colonies. Sterilized pupae were transferred to trays containing 40 L of sterile, *Serratia-*free water and monitored until adult emergence.

Emerged adults were isolated in sterile mesh cages and maintained on 5% sucrose solution. To assess bacterial presence, adults were anesthetized at 24 h post-emergence, surface-sterilized with 70% ethanol (30 seconds), and rinsed in sterile PBS.

Samples from water (centrifuged at 3,000 × *g* for 10 min) as well as homogenized larvae, pupae, and adults were serially diluted in sterile PBS and plated on BHI agar supplemented with kanamycin (100 µg/mL). Plates were incubated at 28°C for 48 h. Due to high expression of the mCherry protein, colonies exhibited distinct red pigmentation visible under a standard binocular microscope (Olympus SZX16). Red colonies were enumerated directly under brightfield illumination without requiring fluorescence filters.

Three independent replicates were conducted. The total sample sizes for each life stage and sample type are summarized in Table 1, which also details the pooling scheme. In brief, we tested a total of 30 second-instar larvae, 30 fourth-instar larvae, 12 pupae, 52 sugar-fed emerged females, 12 sugar-fed emerged males, and three habitat water samples.

### Venereal transmission of *Serratia* AS1-mCherry

Before starting the experimental procedures, mosquito pupae were individually maintained in glass tubes until they emerged as adults. Virgin adult males and females were then collected and placed in separate cages. Fifty virgin males were fed sugar bait solutions containing *Serratia* AS1-mCherry for 36 h. The presence of red abdomens (primarily from red food dye in the sugar bait, with minor contribution from mCherry pigment produced by *Serratia* AS1) confirmed successful bacterial acquisition by males. These males were mixed with 20 virgin 3-day-old females in semi-field cages (24°C–37°C, 22%–89% RH) and allowed to mate. Males and females remained together for 3 days post-mixing until female dissection for CFU quantification in midguts, ovaries, and spermathecae—no separation was performed prior to sampling. To assess bacterial survival post-blood-feeding, a subset of mated females was blood-fed on a rabbit at day 3 post-mixing and dissected 24 h later. Three independent replicates were conducted. The total sample sizes and their distribution per replicate were as follows: 27 sugar-fed females (10, 9, and 8) and 36 blood-fed females (9, 14, and 13). From these specimens, the midgut, ovary, and spermathecae were dissected for testing. Not all tissues could be successfully isolated from every specimen; particularly for blood-fed females, the presence of blood and the expansion of the ovaries reduced the successful dissection rate for ovarian and spermathecal tissues. Consequently, the final number of tissues tested for each type varies, as detailed in Table 1. As a control, three *Serratia* AS1-positive males (one per replicate) were also included.

### Introduction of bacteria through sipping

To test bacterial introduction via sipping, pupae were transferred to larval habitats in confined field conditions (21°C–34°C, 29%–89% RH) with *Serratia* AS1-mCherry added to the breeding water (5 mL of 10^9^ cells/mL in 40 L of water) shortly before adult emergence. Newly emerged adults were maintained on 5% sucrose solution and sampled at 2 days post-emergence for bacterial quantification. A separate group was blood-fed on rabbits on day 3 post-emergence and sampled 24 h later (4 days post-emergence). Surface sterilization and bacterial quantification were performed as described above.

Three independent replicates were conducted. The total sample sizes were 15 sugar-fed females (5 per replicate) and 12 blood-fed females (4 per replicate), resulting in 27 females tested in total. Detailed sample sizes are provided in Table 1.

### Dynamics of *Serratia* AS1-mCherry in larval habitat

To assess the stability of transgenic bacteria in *Anopheles* larval habitats, PE trays (70 × 70 × 17 cm) were used to prevent bacterial dispersion. Each tray was filled with 2 cm–3 cm of garden soil and 40 L of deionized water, then left uncovered outdoors for 20 days to allow natural colonization by *Anopheles* and *Aedes* larvae and aquatic plants. During this pre-experimental period, no *Serratia* AS1-mCherry was introduced, enabling the development of a nutrient-rich, ecologically representative environment. Three treatments evaluated bacterial persistence: water replenished with corn supplementation (5 g corn per tray as nutrient source), water replenishment only (5 L–13 L per tray every 3–5 days to maintain 15 cm water level), and no interventions after initial setup.

At experiment initiation, we added 5 mL of *Serratia* AS1-mCherry suspension (1 × 10^9^ cells/mL) to each tray’s 40 L breeding water (final concentration: 1.25 × 10^5^ cells/mL) and immediately covered them with fine mesh nets within pyramid-shaped cages to prevent further organism entry while maintaining semi-field conditions (22°C–37°C, 15%–95% RH). Bacterial dynamics were monitored twice daily (noon and 8:00 p.m.) until undetectable: 15 days for water-replenished treatments (with or without corn) and 9 days for untreated controls due to water evaporation. A total of 78 water samples were analyzed (two independent samples per day for 39 sampling events across all experimental groups). Water samples were centrifuged, and the pellets were cultured on BHI-kanamycin (100 µg/mL) agar for CFU counting. Environmental parameters (temperature, humidity, pH, total dissolved solids [TDS]) were recorded throughout the assay. Detailed sample sizes are provided in Table 2.

**TABLE 2:** Sample sizes for the bacterial stability assays in breeding water and on sugar baits under different experimental conditions

Assay type	Experimental condition	Environmental variable	Sampling duration (days)	Samples/Replicates per day	Total samples (*n*)
Breeding water	Adding water and corn		15	6	90
	Adding water without corn		15	6	90
	No water or corn added		9	6	54
	Subtotal				234
Sugar bait	Full sunlight	With rainwater	5	3	15
		No rainwater	3	3	9
	Partial sunlight	With rainwater	7	3	21
		No rainwater	6	3	18
	Shade	With rainwater	8	3	24
		No rainwater	6	3	18
	Subtotal				105

### Dynamics of *Serratia* AS1-mCherry on sugar bait

**Fig 2:**
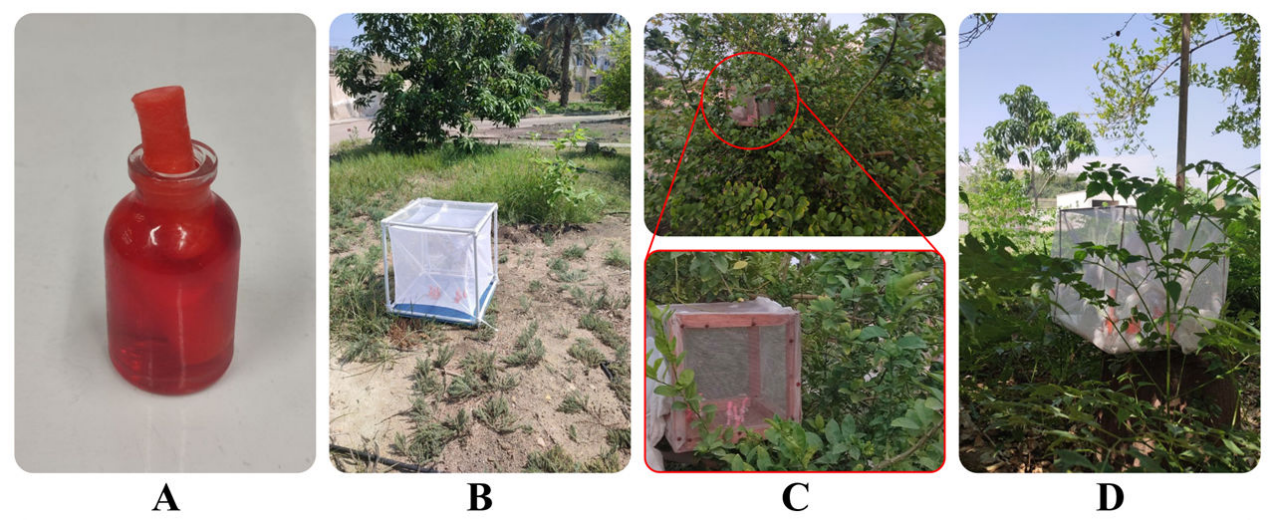
Dynamics of *Serratia* AS1-mCherry in sugar bait under semi-field conditions. (**A**) Experimental setup: 25 mL glass containers containing *Serratia* AS1-mCherry (10^9^ cells/mL) in PBS with 5% (wt/vol) sucrose and 2.5% (wt/vol) food dye, each fitted with a sterile cotton pad. (**B–D**) Containers were placed in protective cages (to restrict bacterial spread) and exposed to different light conditions: (**B**) full sunlight, (**C**) partial sunlight (red line indicates cage position among tree branches), and (**D**) shade.

Bacterial cells were harvested by centrifugation (3,000 × *g*, 10 min), washed twice with sterile PBS, and resuspended in PBS. Sucrose and food dye were then added to achieve final concentrations of 5% (wt/vol) and 2.5% (wt/vol), respectively, with a final cell density of 10^9^ cells/mL in the 25 mL glass container (Fig. 2A). Each container was fitted with a sterile cotton pad. Bacterial presence in the cotton pad was confirmed visually by the development of red coloration (from both the mCherry-tagged bacteria and food dye) and by sucrose diffusion into the pad. The containers were placed in protective cages (to prevent environmental dispersal of bacteria) under three light-exposure conditions: (i) full sunlight, (ii) partial sunlight (elevated 1.5 m on a tree branch), and (iii) shade. Experiments were conducted under semi-field conditions (21°C–36°C, 23%–78% relative humidity) (Fig. 2). To assess the effects of rain or moisture, some solutions were supplemented with 1 mL of rainwater collected at the station, while others were not. Samples were collected daily, and bacterial stability was assessed by culturing on BHI-kanamycin (100 µg/mL) agar. Samples were collected daily until bacterial undetectability by applying gentle pressure to the cotton pad wick with the sampler tips to extract 10 µL of the solution, which was then transferred to microtubes containing 990 µL PBS for serial dilutions.

Sampling duration varied from 3 to 8 days depending on sunlight exposure and rainwater conditions. For each combination of conditions, three independent replicates were maintained, with one sample collected from each replicate daily. Environmental parameters (temperature, humidity) were recorded throughout the experiment. A complete breakdown of sampling durations and total sample sizes for each experimental group is provided in Table 2.

### Isolation and molecular profiling of larval habitat bacteria

This experiment aimed to isolate and characterize bacterial species coexisting with *Serratia* AS1-mCherry in PE trays that served as natural larval habitats for *An. stephensi* larvae. To assess the full diversity of bacteria in the breeding site water, including both kanamycin-resistant and non-resistant strains, no antibiotic selection was applied during initial isolation. During the investigation of this transgenic bacterium’s stability in larval habitat water, a subculture was derived from non-fluorescent bacteria grown on BHI agar alongside *Serratia* AS1-mCherry. The bacteria were separated based on their morphological features (Table 3) and preserved on separate plates at 4°C.

**TABLE 3:** Molecular, morphological properties, and GenBank ID of coexisting bacterial community found in the natural larval habitats of *An. stephensi* in Bandar Abbas, south of Iran, summer 2024^*a*^^,^^*b*^

Species	Phylum	Family	Gram staining	GenBank ID	Previously reported sources		
					Soil	Water	Mosquitoes
*Aeromonas hydrophila*	Pseudomonadota	Aeromonadaceae	Neg-	PQ856157	(44, 45)	(45, 46)	*Culex pipiens* (47) *An. arabiensis* and *An. funestus* (48), *Anopheles* (49)
*Bacillus infantis*	Bacillota	Bacillaceae	Pos+	PQ856158	Garden soil (50, 51), Forest soil (52)	(53) (54)	(*Bacillus* spp.) in *An. subpictus* (55) *An. aconitus* (56)
*Chryseobacterium bernardetii*	Bacteroidota	Weeksellaceae	Neg-	PQ856161	(57)	Other species of this genus (58) but *C. bernardetii*	*Aedes aegypti* (59) *An. sawadwongporni* (60), *An. darlingi* (61, 62), *An. nuneztovari* (62) *Culex quinquefasciatus* (63) *Ae. aegypti* (64), *Ae. albopictus* (65) *An. stephensi* (52)
*Kocuria rhizophila*	Actinomycetota	Micrococcaceae	Pos+	PQ856163	(66)	(67) (68)	*Ae. albopictus* (69) *An. arabiensis* (70) *An. gambiae* and *An. coluzzii* (71)
*Macrococcus equipercicus*	Bacillota	Staphylococcaceae	Pos+	PQ856162	(72)	NA	NA
*Micrococcus luteus*	Actinomycetota	Micrococcaceae	Pos+ to variable	PQ856156	(73)	(74)	*Ae. albopictus* (75) *An. gambiae* (76)
*Proteus vulgaris*	Pseudomonadota	Enterobacteriaceae	Neg-	PQ856160	(77)	Larval habitat (78)	*An. subpictus* (79)
*Staphylococcus hominis*	Bacillota	Staphylococcaceae	Pos+	PQ856159	(80–83)	(84)	*An. gambiae* complex and *An. funestus* (85), *An. arabiensis* (86), *Culex* sp. (87) *An. stephensi* (88)

^*a*^ The report of each species in other sources has been shown. Bacterial strains are arranged in alphabetical order by genus and species names.

^*b*^ NA indicates not reported.

The bacterial specimens were then transferred to the Insect Molecular Biology laboratory at the School of Public Health, Tehran University of Medical Sciences, for further molecular analysis. For molecular characterization, genomic DNA was extracted from the isolated bacteria following the method described by Collins et al. (89). The 16S rRNA gene was amplified using a universal primer pair: forward primer 16suF (5′-GAGTTTGATCCTGGCTCAG-3′) and reverse primer 16suR (5′-GTTACCTTGTTACGACTT-3′), as reported by Weisburg et al. (90), yielding a 1,500 bp fragment. The PCR conditions included an initial denaturation at 94°C for 1 min, followed by 35 cycles of denaturation at 95°C for 30 seconds, annealing at 57.5°C for 40 seconds, and extension at 72°C for 30 seconds (90). A final extension was performed at 72°C for 8 min. The PCR products were then separated on a 1% agarose gel and visualized using a UV transilluminator. The PCR products were purified and subjected to bidirectional Sanger sequencing using the same primers as those employed for PCR (Codon, Iran). The obtained sequences were edited and aligned using BioEdit (version 7.0.9.0), and consensus sequences were subjected to the nucleotide Basic Local Alignment Search Tool to confirm their similarity with sequences deposited in GenBank (https://blast.ncbi.nlm.nih.gov/Blast.cgi). All sequences generated in this study were submitted to the GenBank database (https://www.ncbi.nlm.nih.gov/nucleotide/).

### Statistical analysis

Statistical analyses employed conventional parametric tests and generalized estimating equations (GEE) to address distinct data structures. For cross-sectional data, independent group comparisons utilized Student’s *t*-tests for binary contrasts (e.g., sugar-fed vs blood-fed groups) and one-way analysis of variance (ANOVA) for multi-group comparisons (e.g., across environmental compartments: water, larvae, males, females). *Post hoc* pairwise comparisons following ANOVA were adjusted using Tukey’s honestly significant difference method.

For longitudinal/repeated-measures data (e.g., temporal trends in CFU counts), GEE models with negative binomial distributions and exchangeable correlation structures were implemented to account for temporal autocorrelation, with data clustered by independent replicate (Replicate_ID). For example, in sugar bait experiments, CFU counts were modeled as a function of time (days), sunlight condition (full/partial/shade), and rainwater supplement (yes/no); breeding water analyses modeled CFU counts against time (days), pH, and TDS. GEE model fit and overdispersion were assessed via quasi-likelihood information criterion (QIC), with *post hoc* contrasts (e.g., full sunlight vs shade) tested via Wald tests.

The independent experimental unit (the replication unit) was the biological replicate (*n* = 3 for most experiments). Multiple samples (e.g., pools of individuals) were taken from each replicate. For analyses using *t*-tests and ANOVA, a mean CFU per individual or CFU per pool was first calculated for each replicate separately. These replicate-level mean values were then used to calculate the final overall mean, standard error of the mean (SEM), and for statistical comparisons. This approach prevents pseudoreplication and ensures that the analyses accurately reflect the variation between independent experimental units.

All analyses used a significance threshold of α = 0.05 and were conducted using GraphPad Prism version 5.0 (*t*-tests, ANOVA) and R version 4.3.0 (GEE models, using the geepack package). Figures 7 and 8 (illustrating temporal declines in bacterial stability and variations in pH/TDS) and Fig. 9 (depicting temporal declines in bacterial stability in sugar solution) were generated using Python v.3.12 with Pandas (v.2.2.3) for data handling and Matplotlib (v.3.10.1) and Seaborn (v.0.13.2) for visualization.

## RESULTS

### Vertical transmission of *Serratia* AS1-mCherry in *Anopheles stephensi*

**Fig 3:**
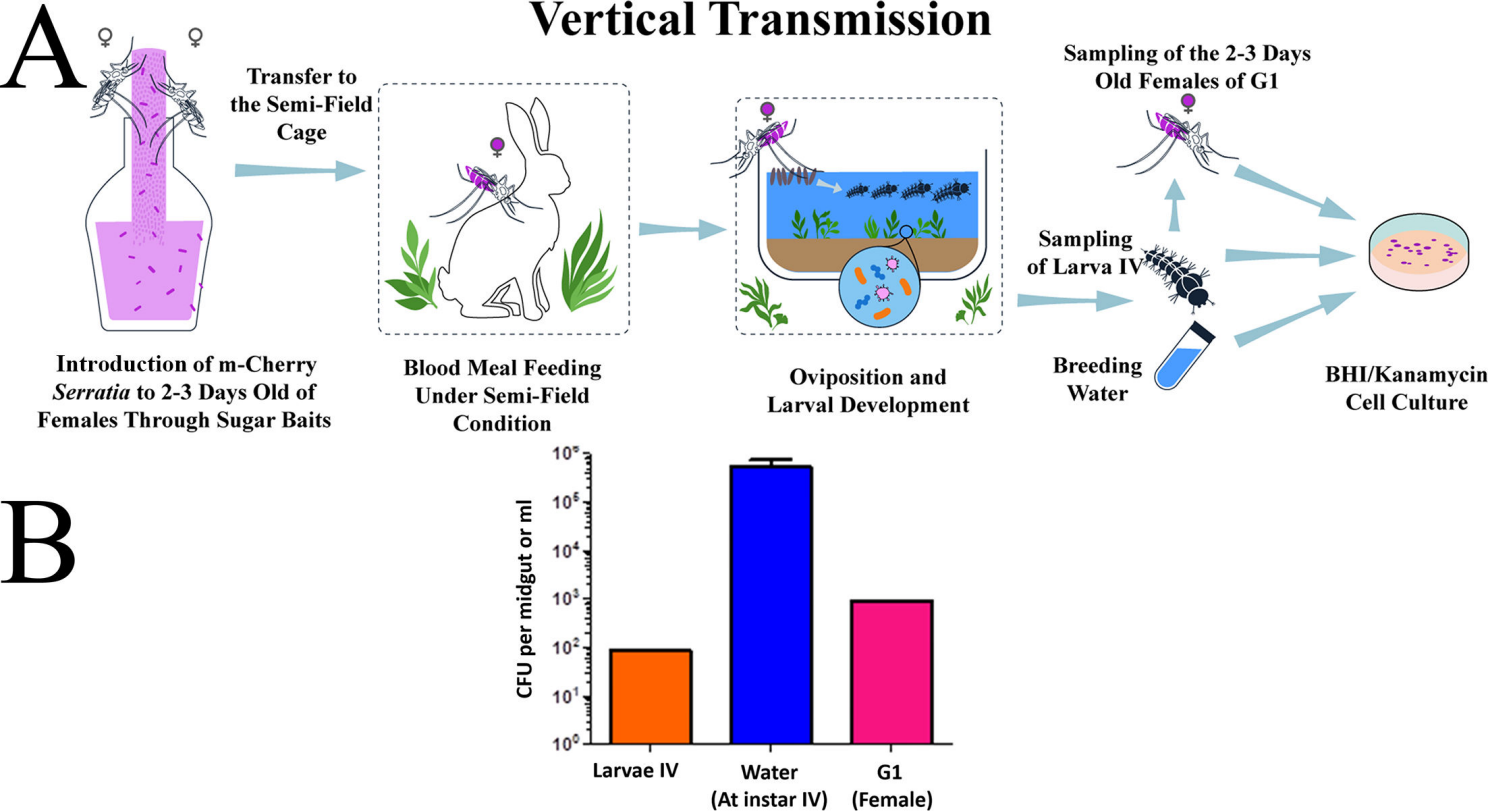
*Serratia* AS1 is vertically transmitted from female *An. stephensi* mosquitoes to larvae, breeding site water, and adults (G1) in confined field conditions**.** (**A**) Fifty 2- to 5-day-old female mosquitoes harboring fluorescent *Serratia* AS1-mCherry were released into large cages under semi-field conditions (21°C–37°C, 15%–95% RH). After blood-feeding on a rabbit, females oviposited in natural soil/water larval habitats. All sampled pool progeny and water exclusively yielded mCherry-fluorescent colonies when plated on BHI-kanamycin during the 12-day monitoring period, confirming sustained detection of plasmid-bearing *Serratia* AS through complete transmission cycles. While offspring abundance (larvae/pupae/adults) could not be quantified due to semi-field challenges, successful reproduction was confirmed. (**B**) Colonization was assessed by sampling the following: 12 pools of fourth-instar larvae (8 pools of 3 and 4 pools of 2), 15 pools of G1 adult females (10 pools of 3 and 5 pools of 2), and 10 mL of breeding water. Bacterial loads were quantified by plating homogenates (larvae/adults) or water samples on BHI-kanamycin (100 µg/mL) agar with exclusive counting of fluorescent colonies to track plasmid-bearing *Serratia* AS1. Data represent three independent replicates (mean ± SEM), with experiments repeated three times with consistent results.

Figure 3 demonstrates efficient transmission of *Serratia* AS1-mCherry from female *An. stephensi* mosquitoes to their offspring and breeding water under confined field conditions. The bacteria were detected in 100% of the progeny pools examined (larvae: 12/12 pools; G1 females: 15/15 pools). Mean bacterial loads were 87.14 ± 11.76 CFU/larva in larvae and 906.66 ± 45.45 CFU/female gut in G1 females. Breeding water maintained high concentrations (1.04 × 10^6^ ± 6.81 × 10^4^ CFU/mL) of fluorescent colonies, confirming environmental persistence of the plasmid-bearing strain during the observation period. Notably, all detected colonies exhibited mCherry fluorescence at this stage (days 0–12), with no evidence of plasmid loss during initial transmission events.

The quantitative differences in bacterial loads reflect compartment-specific dynamics: water functioned as both transmission medium and reservoir, while larval and adult tissues showed active colonization. The universal infection of all pooled offspring (100%) alongside persistent environmental detection establishes concurrent vertical and environmental transmission pathways. These results confirm stable maintenance of the mCherry plasmid through complete mosquito life cycles under field conditions, though longer-term persistence would require monitoring plasmid stability beyond 12 days (as addressed in Fig. 7).

### Venereal transmission of *Serratia* AS1-mCherry in *An. stephensi*

**Fig 4:**
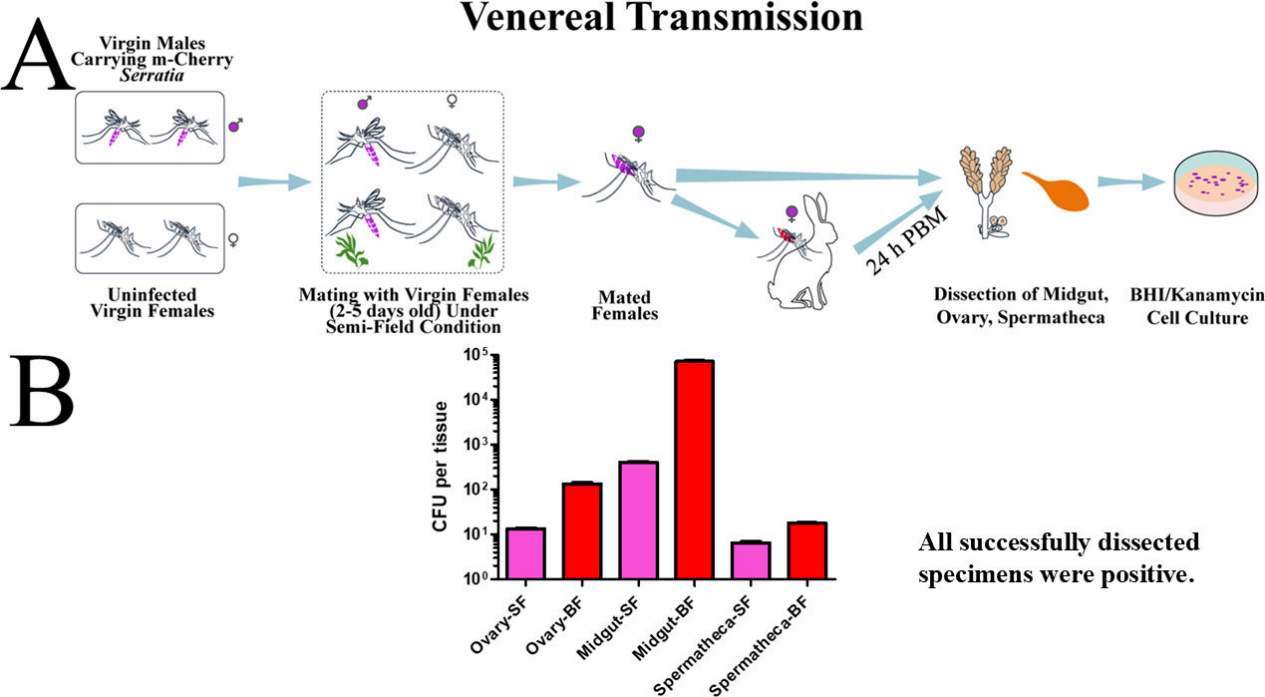
*Serratia* AS1 venereal transmission dynamics in *Anopheles stephensi* under semi-field conditions. (**A**) Virgin male mosquitoes were provisioned with 5 mL of 10^9^ CFU/mL fluorescent *Serratia* AS1-mCherry in a 5% sucrose solution for 36 h before mating with age-matched virgin females. Bacterial isolations from female midguts and reproductive tracts 72 h post-mating yielded exclusively mCherry-expressing colonies on selective media. Plasmid-bearing *Serratia* AS1 was detected in both sugar-fed (SF) and blood-fed (BF, 24 h post-blood meal) females, confirming successful sexual transmission. (**B**) At 72 h post-mating, females were divided into blood-fed and sugar-fed cohorts. Quantitative analysis revealed significantly higher bacterial loads in blood-fed females across all tissues (midguts: *P* < 0.0001; ovaries: *P* < 0.0001; spermatheca: *P* < 0.0001; unpaired Student’s *t*-test, *n* = 3 biological replicates per group). Data are presented as mean ± SEM. Three independent replicate experiments confirmed efficient venereal transmission, with fluorescent colonies detected in 100% of successfully dissected samples across all replicates.

Figure 4 demonstrates successful venereal transmission of *Serratia* AS1-mCherry in *An. stephensi* under semi-field conditions. Orally infected virgin males efficiently transmitted the plasmid-bearing bacteria to naive females during mating. The bacterium was recovered from all successfully dissected female reproductive tissues and midguts. Specifically, the mean bacterial loads were 13.4 ± 0.4 CFU in ovaries (*n* = 21), 6.6 ± 0.2 CFU in the spermatheca (*n* = 20), and 402.6 ± 6.4 CFU in the midgut (*n* = 27). This systemic dissemination of fluorescent bacteria is consistent with *Serratia’*s known capacity to cross epithelial barriers (5), while confirming maintained plasmid integrity during sexual transmission.

Blood-fed females exhibited significantly higher bacterial loads than sugar-fed controls across all examined tissues. The most substantial increase was a 175.8-fold change in the midgut (*t*(61) = 14.31, *P* < 0.0001), followed by the ovaries (10-fold; *t*(35) = 14.52, *P* < 0.0001) and the spermatheca (2.7-fold; *t*(25) = 17.92, *P* < 0.0001). Statistical analysis was performed using an unpaired Student’s *t*-test (*n* = 3 biological replicates per group). Sample sizes for dissections were as follows: midgut (blood-fed: *n* = 36, sugar-fed: *n* = 27), ovaries (blood-fed: *n* = 16, sugar-fed: *n* = 21), and spermatheca (blood-fed: *n* = 7, sugar-fed: *n* = 20). The enhanced proliferation of mCherry-positive *Serratia* suggests blood meals create a favorable microenvironment for plasmid-bearing bacteria, potentially through nutrient enrichment (e.g., hemoglobin-derived iron) and transient immune suppression during blood digestion. These results demonstrate that the mCherry plasmid remains stable through both male acquisition and subsequent venereal transmission events.

### Transstadial transmission of *Serratia* AS1-mCherry

**Fig 5:**
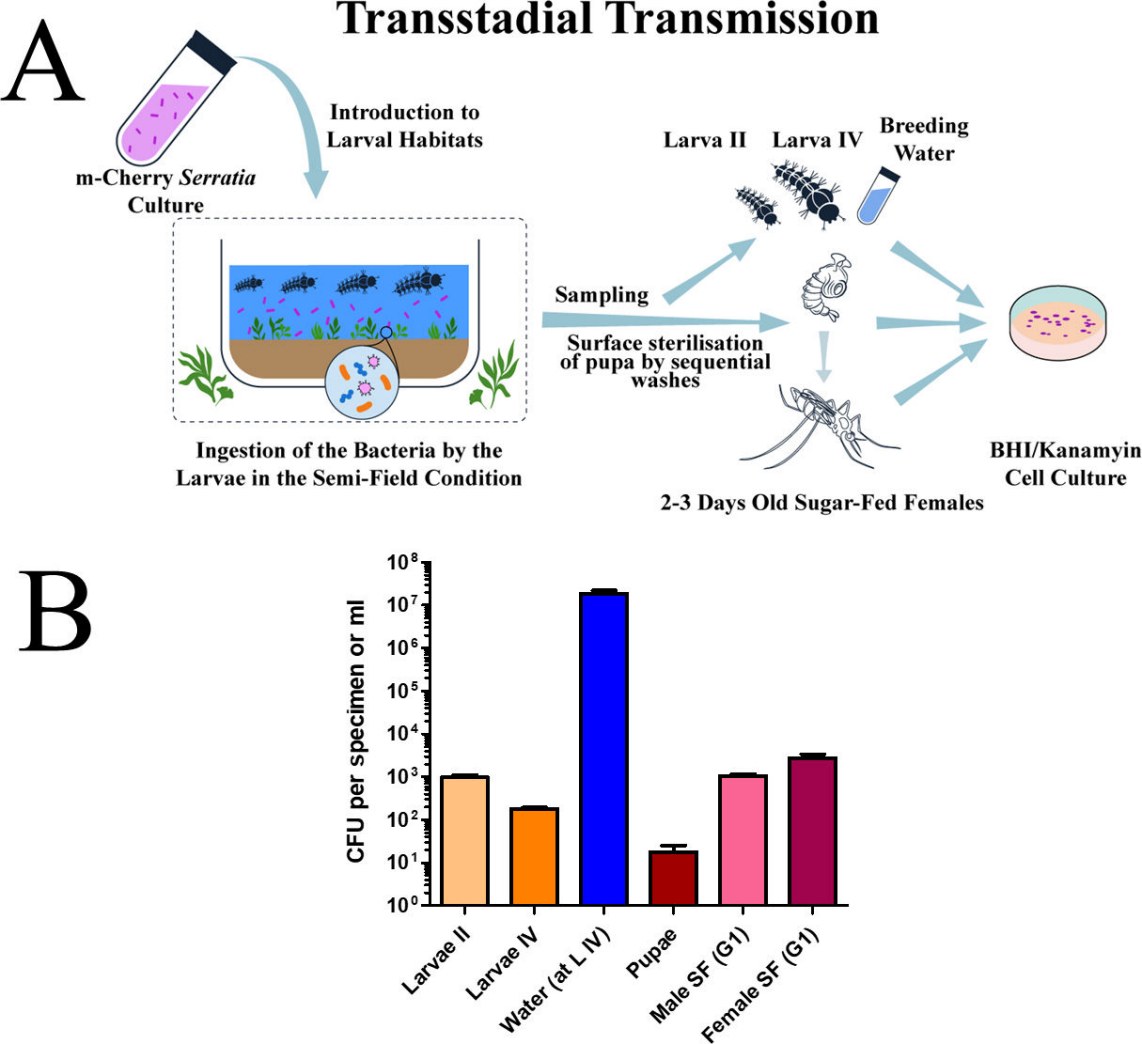
Transstadial transmission of *Serratia* AS1-mCherry in *Anopheles stephensi* mosquitoes. (**A**) Larvae were exposed to *Serratia* AS1-mCherry (5 mL of 10^9^ CFU/mL) added to breeding water (40 L) under semi-field conditions (22°–37°C, 15%–95% RH). Bacterial persistence was tracked through complete metamorphosis. mCherry-expressing colonies were exclusively recovered on selective media from the following pooled samples: 30 second-instar larvae (II) (8 pools of 3 and 3 pools of 2 larvae each), 30 fourth-instar larvae (IV) (8 pools of 3 and 3 pools of 2 larvae each), 12 pupae (no pooling), 12 sugar-fed (SF) male adults (G1 generation; 4 pools of 3 adults each), 52 sugar-fed (SF) female adults (G1 generation; 14 pools of 3 and 5 pools of 2 adults each), and three breeding water samples (10 mL per replicate). (**B**) Bacterial loads were quantified by plating homogenates (or water samples) on BHI agar containing kanamycin (100 µg/mL), with fluorescent colony counts confirming the presence of plasmid-bearing *Serratia* AS1. Data represent mean ± SEM from three independent replicates. The consistent detection of fluorescent colonies across all developmental stages demonstrates uninterrupted transstadial transmission. Quantitative analysis revealed a significant reduction in bacterial load between the larval stages and the pupal stage (second-instar vs pupa: *P* < 0.05; fourth-instar vs pupa: *P* < 0.05; unpaired *t*-test, *n* = 3 biological replicates). No other inter-stage comparisons were statistically significant, likely due to high biological variability.

As shown in Fig. 5, *Serratia* AS1-mCherry demonstrated complete transstadial transmission from larvae to adult *An. stephensi* while maintaining plasmid stability. Larval breeding water was supplemented with 5 mL × 10^9^ CFU/mL of fluorescent *Serratia* AS1-mCherry, resulting in successful colonization across all developmental stages. While we cannot exclude environmental bacterial acquisition during larval stages due to sterilization challenges, surface-sterilized pupae confirmed endogenous transstadial transmission. mCherry-positive colonies were consistently recovered from all stages (mean ± SEM): second-instar larvae (1,117.17 ± 274.99 CFU/larva; *n* = 11 pools), fourth-instar larvae (182.49 ± 32.7 CFU/larva; *n* = 11 pools), surface-sterilized pupae (13.4 ± 5.2 CFU/pupa; *n* = 12 individuals), and adults (males 1,145.55 ± 282.97 CFU, *n* = 4 pools; females: 3,067.59 ± 1,587.48 CFU, *n* = 19 pools).

This demonstrates at minimum (i) successful bacterial persistence through complete metamorphosis (pupae-to-adult) and (ii) that larval-stage transmission may include environmental contributions. The uniform bacterial counts between sexes (*P* > 0.05) and across life stages indicate both efficient transstadial transmission and stable maintenance of the mCherry plasmid throughout mosquito development. These findings demonstrate that the genetically modified phenotype remains intact during the critical larval-to-adult transition, a key requirement for field applications.

### Introduction of *Serratia* AS1-mCherry to newly emerged adults (sipping)

**Fig 6:**
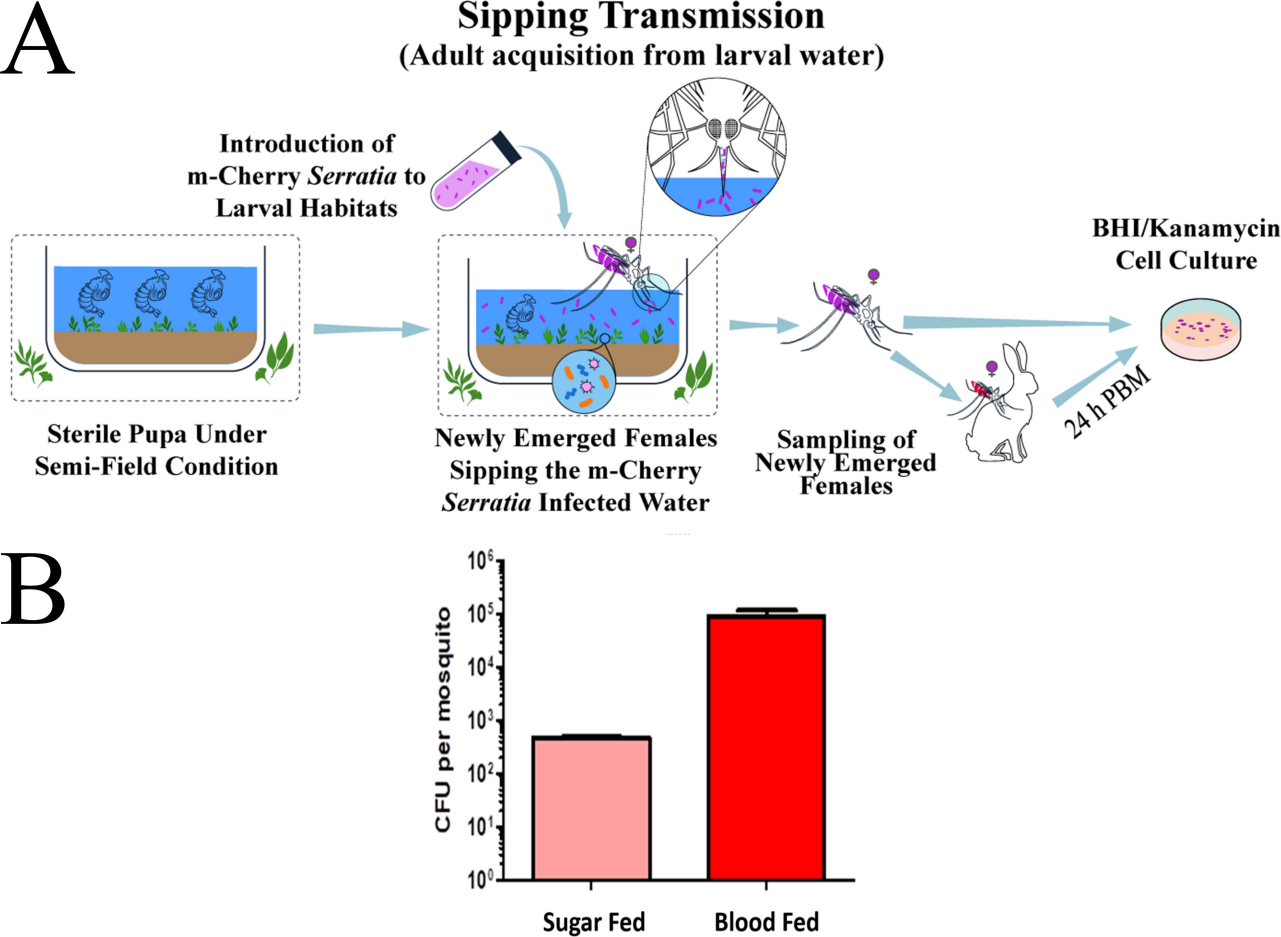
Introduction of mCherry**-***Serratia* AS1 to newly emerged adults of *An. stephensi* via breeding water (sipping). (**A**) Free pupae were transferred to larval habitats (21°C–34°C, 29%–89% RH) containing 40 L water inoculated with 5 mL of 10^9^ cells/mL *Serratia* AS1-mCherry prior to adult emergence. Emerged adult females were separated into two dietary groups: those maintained on 5% sucrose (*n* = 15) and those given rabbit blood meals (*n* = 12). All recovered colonies (plated on BHI-kanamycin [100 µg/mL] agar) maintained mCherry fluorescence, confirming plasmid stability during both environmental exposure and host colonization. (**B**) Quantitative analysis revealed that blood-fed females harbored significantly higher bacterial loads than sugar-fed females, suggesting a dietary effect on bacterial colonization (*P* = 0.037, unpaired Student’s *t*-test, Welch’s correction, *t*(2.1) = 4.85, *n* = 3 biological replicates per group). This finding is particularly significant as it shows the mCherry plasmid remains stable during both environmental persistence in water and subsequent host colonization—two critical phases for field applications.

In Fig. 6, we assessed the introduction of plasmid-bearing *Serratia* AS1-mCherry to newly emerged *An. stephensi* adults via breeding water (sipping). The transgenic bacteria (5 mL of 10^9^ CFU/mL) were added to 40 L of breeding water immediately before adult emergence. All specimens positive for *Serratia* AS1-mCherry exhibited fluorescent colonies, confirming successful uptake and stable plasmid maintenance during sipping-mediated colonization. The mean bacterial load was 478.7 ± 20.7 CFU/female in sugar-fed specimens and 93,600 ± 19,335 CFU/female in blood-fed specimens. The consistent detection of fluorescent colonies across replicates confirms the reliable transfer of the genetically modified bacteria through natural drinking behavior. Quantitative analysis revealed that blood-fed females harbored significantly higher bacterial loads than sugar-fed females, suggesting a dietary effect on bacterial colonization (*P* < 0.05, unpaired Student’s *t*-test, *n* = 3 biological replicates per group). This finding is particularly significant as it shows the mCherry plasmid remains stable during both environmental persistence in water and subsequent host colonization—two critical phases for field applications.

### Dynamics of *Serratia* AS1-mCherry in larval breeding habitats

**Fig 7:**
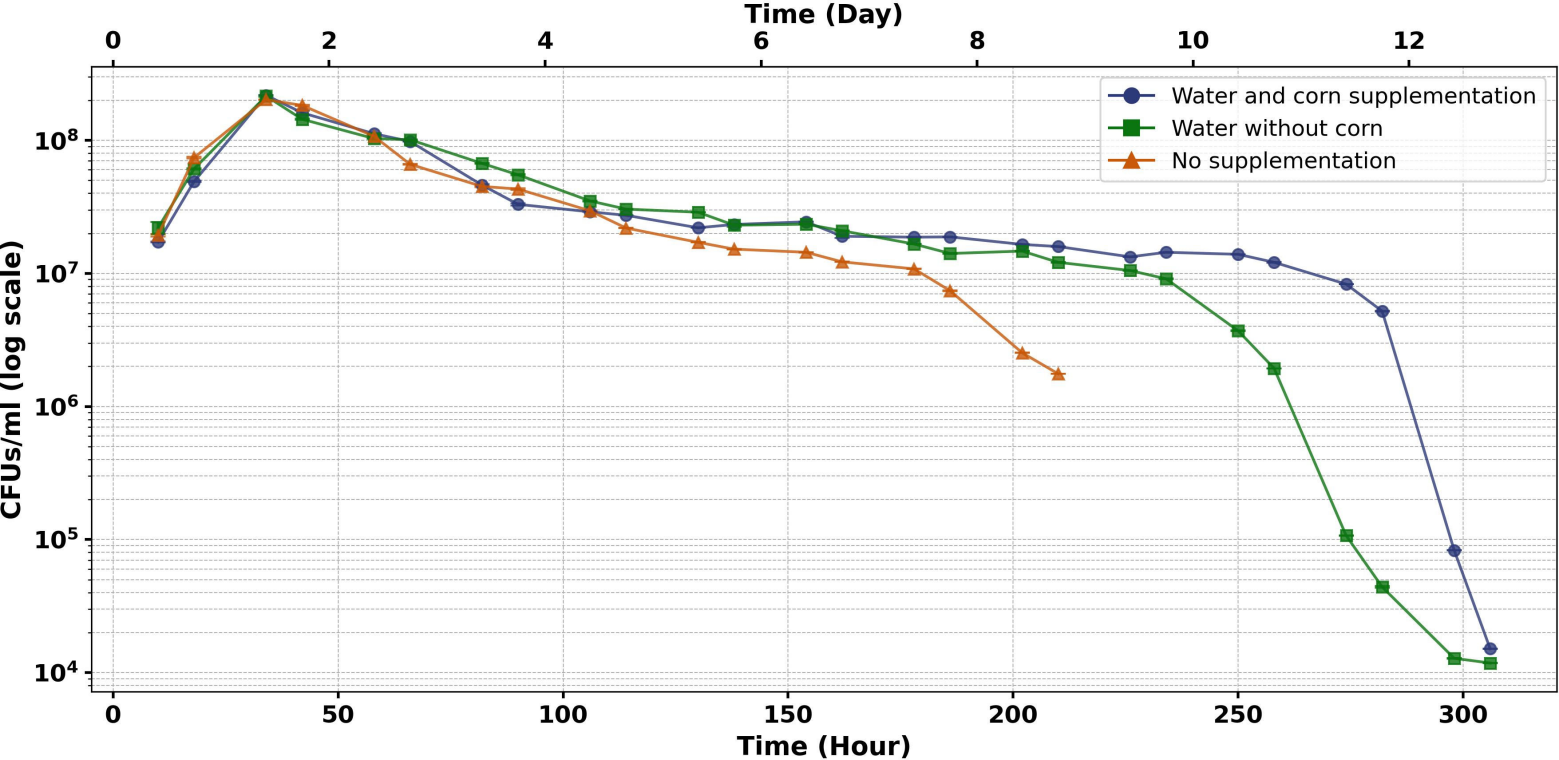
Temporal dynamics of plasmid-bearing *Serratia* AS1-mCherry in larval breeding water systems. The experimental setup consisted of breeding trays with 2 cm–3 cm garden soil and 40 L deionized water, inoculated with 5 mL × 10^9^ cells/mL fluorescent *Serratia* AS1-mCherry. Systems were maintained under field conditions (22°C–37°C, 15%–95% RH; pH 7.72–9.14, TDS 3,340 ppm–9,900 ppm) for 20 days. Twice-daily fluorescent colony counts (12:00/20:00) via plating on BHI-kanamycin (100 µg/mL) revealed that corn-supplemented systems sustained >5.2 × 10^6^ CFU/mL of mCherry-expressing bacteria through day 12, while unsupplemented controls showed complete plasmid loss by day 9. All systems reached undetectable fluorescent colony counts by day 14 (water + corn > water-only > unsupplemented controls), demonstrating consistent plasmid clearance within 2 weeks under all tested conditions.

Persistence of *Serratia* AS1-mCherry varied significantly between treatment groups (Fig. 7). Water + corn supplementation sustained >5.2 × 10^6^ CFU/mL of plasmid-bearing bacteria through day 12, while water-only and unsupplemented controls exhibited rapid decline in detectable fluorescent colonies. Fluorescent bacterial counts declined at a rate of 38% daily (β = −0.48 ± 0.02, *P* < 0.001), with nutrient-supplemented groups maintaining detectable mCherry-expressing colonies for up to 14 days compared to only 9 days in untreated controls.

A negative binomial GEE model (QIC = 475.1) identified treatment type as the primary predictor of bacterial persistence, with additional effects from sampling time (22% higher evening counts; β = 0.20 ± 0.03) and total dissolved solids (11% viability reduction per 1,000 ppm; β = −0.12 ± 0.01). Notably, pH showed no significant effect on fluorescent colony detection (*P* = 0.18).

**Fig 8:**
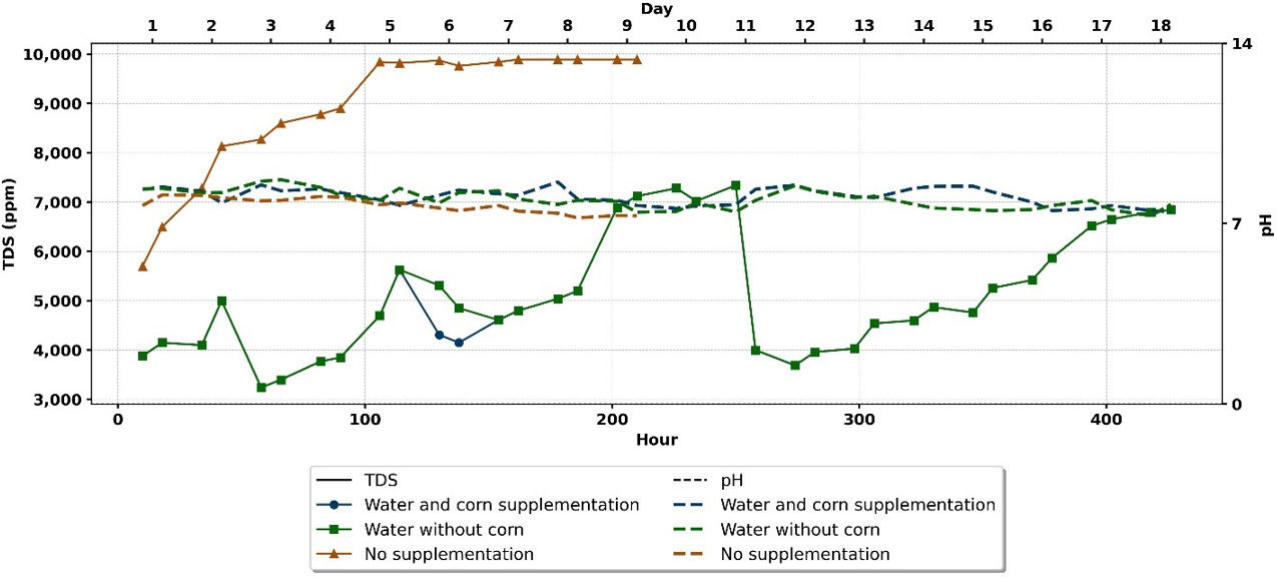
Temporal dynamics of pH and TDS in larval breeding water systems. The experimental setup consisted of breeding trays containing 2 cm–3 cm of garden soil and 40 L of deionized water, inoculated with *Serratia* AS1-mCherry (5 mL × 10^9^ cells/mL). Systems were maintained under field conditions (22°C–37°C, 15%–95% RH) for 20 days. pH and TDS were measured across three treatment regimens: water + corn supplementation, water-only, and unsupplemented controls. Data are pooled from three independent experimental runs (*n* = 3 per treatment).

These biological trends occurred against a backdrop of dynamic abiotic conditions (Fig. 8), which documented daily pH and TDS fluctuations under field conditions (22°C–37°C, 15%–95% RH) for 20 days across three treatment regimens. Data represent three independent replicates per treatment.

Beyond day 14, only non-fluorescent colonies persisted, confirming plasmid instability under prolonged field exposure. Together, these results demonstrate that while nutrient availability and diurnal fluctuations regulate total bacterial persistence (Fig. 7), the maintenance of the genetically modified phenotype operates within a narrower temporal window that is critical for paratransgenesis applications.

**Fig 9:**
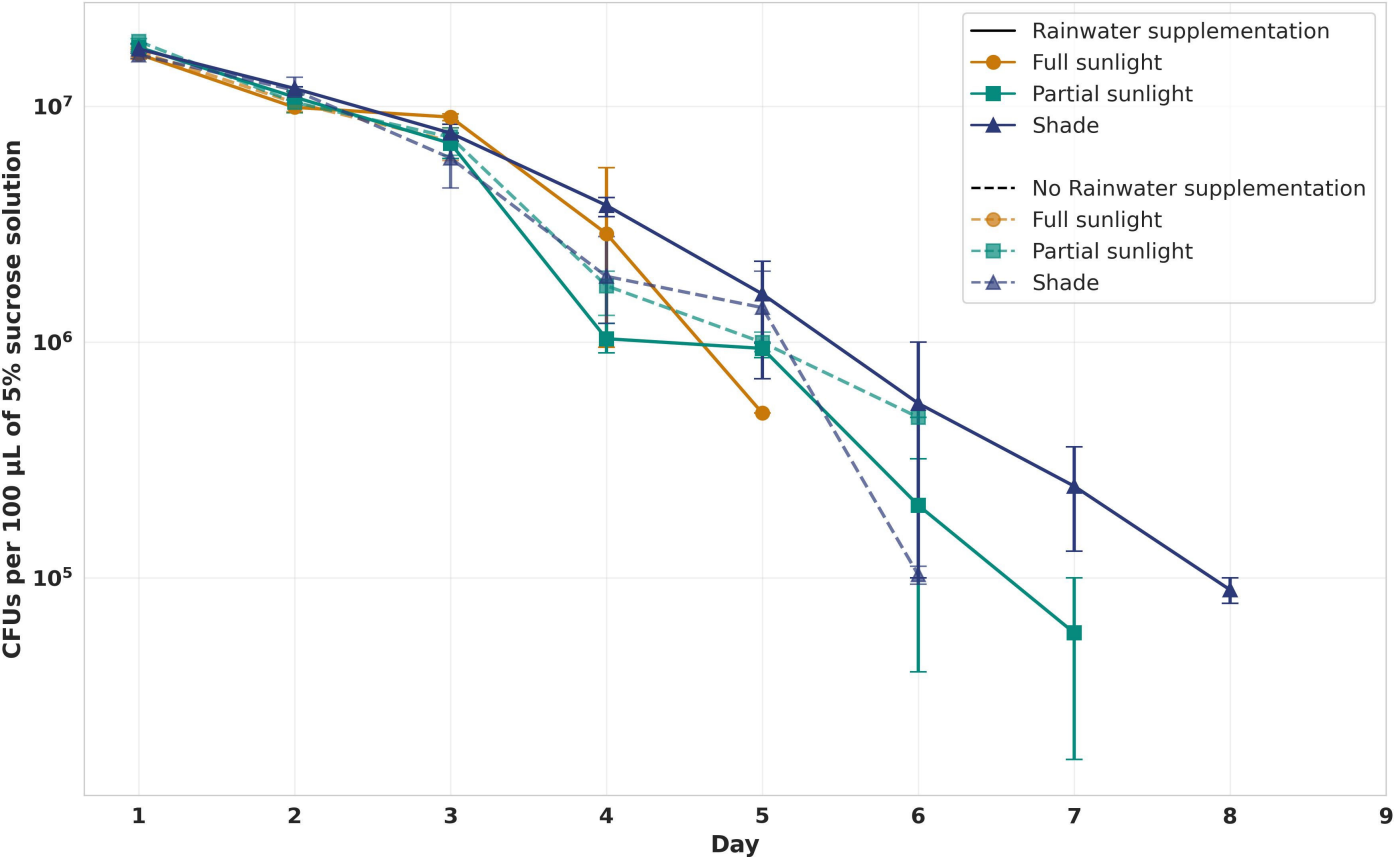
Dynamics of *Serratia* AS1-mCherry in sugar solution embedded in cotton pads under confined field conditions. Solutions (10^9^ cells/mL in 5% sucrose + 2.5% food-grade dye) were maintained in cotton pads with PBS under three environments: full sunlight (orange), partial sunlight (green; 1.5 m elevation), and shade (blue), with/without daily 1 mL rainwater supplementation (solid/dashed lines; *n* = 3 replicates). GEE-adjusted trendlines confirmed differences in decay rates: full sunlight (−0.68 ± 0.05 logCFU/day, *P* < 0.001) vs shade (−0.49 ± 0.04 logCFU/day, *P* < 0.001; Wald test: χ² = 12.7, *P* = 0.002). Rainwater supplementation had no significant effect (*P* = 0.38). Field conditions: 21°C–36°C, 23%–78% RH. CFUs were quantified daily via BHI-kanamycin (100 µg/mL) (selection for the mCherry plasmid) plating of 10 µL wick extracts. Three experimental repeats yielded consistent results (QIC = 152.3).

The environmental persistence of *Serratia* AS1-mCherry was quantified in sugar solutions under full sunlight, partial sunlight (1.5 m elevation), and shade (Fig. 9). GEE revealed significant effects of time and sunlight exposure on detectable fluorescent colony counts (all *P* < 0.001). Fluorescent bacteria persisted longest in shaded conditions (8 days with rainwater; 6 days without), exhibiting a 28% slower decline rate than full sunlight (β = 0.25 ± 0.07, *P* = 0.002). Partial sunlight showed intermediate fluorescence persistence (7 days with rainwater; 6 days without), with no significant rainwater effect (*P* = 0.38). Full sunlight yielded the shortest detectable fluorescence duration (5 days with rainwater; 3 days without) and fastest decay (β = −0.68 ± 0.05 /day). Comparative analysis with breeding water experiments confirmed that plasmid-containing *Serratia* AS1 (detected as mCherry-expressing colonies) persisted longer in aquatic habitats than in sugar solutions (*P* < 0.001), supporting their utility for field delivery. Shade significantly extended the detectable presence of plasmid-containing bacteria in sugar solutions—a critical factor for application design.

### Bacterial species in breeding site water

BHI agar culture and 16S rRNA sequencing of water samples from breeding sites in the PE trays revealed the presence of eight bacterial species including *Micrococcus luteus*, *Macrococcus equipercicus, Proteus vulgaris*, *Aeromonas hydrophila*, *Bacillus infantis*, *Staphylococcus hominis*, *Chryseobacterium bernardetii,* and *Kocuria rhizophila* (Table 3). *Aeromonas hydrophila*, belonging to the family *Aeromonadaceae* of the Pseudomonadota phylum, was the most prevalent species in the breeding sites. Gram-positive bacteria were more diverse, but Gram-negative bacteria were more abundant.

## DISCUSSION

Malaria continues to pose a significant global health burden, with existing control strategies increasingly hindered by parasite resistance to drugs and mosquito resistance to insecticides (4, 38). The urgent need for innovative solutions has driven interest in paratransgenesis approaches using *Serratia* AS1 (5, 91, 92), which this study evaluated under semi-field conditions in a malaria-endemic region. Our findings address the critical challenge of delivering genetically modified bacteria to wild mosquito populations while advancing both the potential and practical considerations for operational deployment.

The ecological compatibility of *Serratia* AS1 was demonstrated through its coexistence with native microbiota, including immune-modulating bacteria like *Micrococcus luteus* (93, 94). These interactions suggest minimal disruption to microbial communities while potentially bolstering vector immune responses—findings consistent with established relationships between microbiome composition and vectorial capacity (95–98). Notably, *Serratia* AS1 maintained plasmid stability (mCherry expression confirmed by fluorescence screening) for up to 13 days in water-based systems and 5–8 days in sugar baits under variable field conditions (pH 7.72–9.14; TDS 3,340–9,900 ppm), covering the critical window for mosquito development. The ability of *Serratia* AS1 to disseminate across mosquito life stages—via transstadial, venereal, vertical, and sipping transmission—further underscores its potential for widespread establishment within vector populations, aligning with previous findings on its minimal fitness cost to mosquitoes (5, 25, 28, 91, 92, 99).

Complete vertical transmission (100%) of *Serratia* AS1-mCherry likely occurs via transovum transmission, where bacteria colonize egg surfaces during oviposition (5). This is supported by both direct evidence of *Serratia* adhesion to egg surfaces (5, 91, 99) and the sustained high bacterial loads in breeding water (1.04 × 10^6^ CFU/mL) from larval defecation and environmental proliferation. This environmental persistence enables a dual transmission strategy, contrasting with obligate intracellular symbionts like *Wolbachia* (97). Despite larval sterilization attempts, larval feeding and excretion persistently reintroduced *Serratia* AS1-mCherry into the water, complicating differentiation between true transstadial transmission and environmental reacquisition.

Water-based delivery supported significantly longer detection of plasmid-bearing Serratia AS1 (9–13 days; Fig. 7) than sugar bait applications (5–8 days; Fig. 8). Two key factors emerged: (i) nutrient-rich aquatic environments enhanced by corn supplementation prolonged detectable mCherry-expressing colonies (13 vs 9 days), and (ii) shaded conditions extended persistence of fluorescent bacteria in sugar baits (8 vs 3–5 days in full sunlight).

The abrupt CFU decline between days 12 and 14 revealed environmental vulnerabilities. A rain event elevated pH from 7.7 to 8.8 through carbonate buffering, exceeding *Serratia*’s optimal range, while ammonia influx from industrial/agricultural dust likely exacerbated toxicity (100). Nutrient exhaustion accelerated the decline, particularly in unsupplemented groups, with group 3 (no additions) showing the most rapid collapse (day 9) due to hyperosmotic stress (TDS ≥9,900 ppm) and complete nutrient depletion.

These dynamics inform critical field implementation considerations. The predictable disappearance of fluorescent colonies, followed by the emergence of plasmid-free colonies, demonstrates controlled phenotypic loss (5, 92), addressing regulatory concerns (101). Importantly, rigorous safety assessments showed negligible horizontal gene transfer risk (<1.5 × 10⁻⁶) among >1.5 million screened colonies, with no detectable plasmid transmission to environmental or mosquito-associated bacteria, including conjugation-competent *Escherichia coli* and *Pantoea agglomerans* (92). Plasmid loss was rapid, occurring within ~130 bacterial generations *in vitro* and within three mosquito generations *in vivo* (92), ensuring reversion to wild-type strains and minimizing environmental persistence of engineered traits. The bacterium imposed no fitness costs on mosquitoes or non-target species (25, 98, 102, 103), though implementation challenges remain regarding reapplication frequency and larval transmission validation.

The integration of *Serratia* AS1 into existing vector control strategies, such as larvicides, ITNs, or IRS, offers synergistic potential, although interactions between interventions warrant further exploration. Scalability will depend not only on delivery optimization but also on securing regulatory approval and public acceptance through robust risk assessment and community engagement.

The safety of *Serratia* AS1-mCherry for humans and non-target organisms remains a critical consideration. While genome analyses of related *Serratia* strains identify virulence factors like serralysins, hemolysins, and iron-uptake systems (21), *Serratia A*S1’s intrinsic susceptibility to common antibiotics (kanamycin, streptomycin, tetracycline) (92) and rapid loss of plasmid-borne resistance (5, 92) provide dual biocontainment. Empirical studies have shown no adverse effects on mosquitoes (5, 25, 28, 99) or non-target species, including native bees (*Partamona helleri*) (101), rodents (*Rhombomys opimus*) (102), and plants (*Haloxylon persicum*) (103). Moreover, over a decade of laboratory and semi-field work with high bacterial exposures has yielded no reported infections among researchers (5, 25, 26, 28, 99, 102, 103). These findings, while anecdotal, align with the absence of documented human pathogenicity for mosquito-derived *Serratia* strains, supporting the low-risk profile of *Serratia* AS1 for field application. Nevertheless, comprehensive pathogenicity studies in mammalian models remain essential to fully validate biosafety.

### Conclusion

*Serratia* AS1 demonstrates a balanced profile of efficacy and safety, with successful transmission via vertical, venereal, transstadial, and sipping routes, and plasmid loss supporting biocontainment. Field deployment should prioritize larval habitat treatment and the placement of nutrient-supplemented, shaded sugar baits to target adults. Future research should focus on optimizing formulations with extended-release components, determining reapplication timing, identifying effective effector molecules, assessing mammalian biosafety, and fostering community acceptance. Addressing these translational challenges will be essential to unlocking the full potential of this strategy for sustainable malaria control, particularly in regions with high insecticide resistance.

## Data Availability

The sequence data obtained in this study are available in the GenBank database under accession numbers PQ856156 to PQ856163.
